# Thioredoxin Domain Containing 5 Suppression Elicits Serum Amyloid A-Containing High-Density Lipoproteins

**DOI:** 10.3390/biomedicines10030709

**Published:** 2022-03-18

**Authors:** Javier Sánchez-Marco, Roberto Martínez-Beamonte, Alicia De Diego, Tania Herrero-Continente, Cristina Barranquero, Carmen Arnal, Joaquín Surra, María A. Navarro, Jesús Osada

**Affiliations:** 1 Departamento de Bioquímica y Biología Molecular y Celular, Facultad de Veterinaria, Instituto de Investigación Sanitaria de Aragón-Universidad de Zaragoza, E-50013 Zaragoza, Spain; javiersanchezmarc@gmail.com (J.S.-M.); romartin@unizar.es (R.M.-B.); taniaherrero1992@gmail.com (T.H.-C.); angelesn@unizar.es (M.A.N.); 2 Instituto Agroalimentario de Aragón, CITA-Universidad de Zaragoza, E-50013 Zaragoza, Spain; cbarranq@unizar.es (C.B.); arnal@unizar.es (C.A.); jsurra@unizar.es (J.S.); 3 CIBER de Fisiopatología de la Obesidad y Nutrición, Instituto de Salud Carlos III, E-28029 Madrid, Spain; 4 Instituto de Investigación Sanitaria de Aragón (IISA), Universidad de Zaragoza, E-50013 Zaragoza, Spain; adediego.iacs@aragon.es; 5 Departamento de Patología Animal, Facultad de Veterinaria, Instituto de Investigación Sanitaria de Aragón-Universidad de Zaragoza, E-50013 Zaragoza, Spain; 6 Departamento de Producción Animal y Ciencia de los Alimentos, Escuela Politécnica de Huesca, Instituto de Investigación Sanitaria de Aragón-Universidad de Zaragoza, E-22071 Huesca, Spain

**Keywords:** thioredoxin domain containing 5, TXNDC5, serum amyloid, HDL, SAA, *Saa1*, *Saa2*, liver, *Txndc5*-deficient mice, RNAseq

## Abstract

Thioredoxin domain containing 5 (TXNDC5) is a protein disulfide isomerase involved in several diseases related to oxidative stress, energy metabolism and cellular inflammation. In a previous manuscript, a negative association between fatty liver development and hepatic *Txndc5* expression was observed. To study the role of TXNDC5 in the liver, we generated *Txndc5*-deficient mice. The absence of the protein caused an increased metabolic need to gain weight along with a bigger and fatter liver. RNAseq was performed to elucidate the putative mechanisms, showing a substantial liver overexpression of serum amyloid genes (*Saa1*, *Saa2*) with no changes in hepatic protein, but discrete plasma augmentation by the gene inactivation. Higher levels of malonyldialdehyde, apolipoprotein A1 and platelet activating factor-aryl esterase activity were also found in serum from *Txndc5*-deficient mice. However, no difference in the distribution of high-density lipoproteins (HDL)-mayor components and SAA was found between groups, and even the reactive oxygen species decreased in HDL coming from *Txndc5*-deficient mice. These results confirm the relation of this gene with hepatic steatosis and with a fasting metabolic derive remedying an acute phase response. Likewise, they pose a new role in modulating the nature of HDL particles, and SAA-containing HDL particles are not particularly oxidized.

## 1. Introduction

The transcriptome is the complete set of transcripts in the cell, including mRNA (mRNA), non-coding RNAs (ncRNA) and small RNA [1]. In mammals only, around 20,000 of these transcripts are translated, but alternative splicing generates an even larger number of different proteins [2,3]. Approximately a third of the proteins are being transported through the endoplasmic reticulum (ER), an organelle specialized in protein homeostasis, folding and assembly, in which the oxidative environment maintained by the glutathione balance is crucial to the correct formation of disulfide bonds [4].

The protein disulfide isomerase family (PDIs) and several chaperones work together to reduce the risk of misfolding and aggregation in the ER [5,6]. PDIs contain at least one thioredoxin (TRX)-like domain that catalyze the protein disulfide isomerase and redox activity [6,7]. One of these PDI, thioredoxin domain containing protein 5 (TXNDC5), also known as resident endoplasmic reticulum 46 (Erp46) or PDI15, contains three TRX domain which show a CXXC catalytic [8] sequence involved in redox and disulfide isomerase activities, followed by a C-terminal KDEL sequence that retains it in the ER [9].

Several biological functions of TXNDC5 have been proposed, including anti-oxidation, angiogenesis promotion, energy metabolism and involvement in cellular inflammation among others [10,11,12]. According to that, several studies have proved that a high expression of Txndc5 is linked with high resistance tumors including hepatocellular, cervical and gastric carcinome, prostate and renal cell adenocarcinoma, and rhabdomyosarcoma and colorectal cancer [10,11,13,14,15,16,17,18,19,20]. Lastly, it is involved in other diseases correlated with oxidative tissues such as rheumatoid arthritis (RA), diabetes, and non-alcoholic fatty liver [12,21,22,23,24].

In a previous manuscript from the group, TXNDC5 was found negatively associated with the degree of fatty liver development, following squalene administration [8]. Squalene is the main hydrocarbon present in extra virgin olive oil and has been postulated to be one of the main components to modulate the known anti-hepatic steatosis effect of the Mediterranean diet in *Apoe*-deficient mice [25,26,27]. Chronic liver disease is a major cause of morbidity and mortality in which the progression is characterized by an ongoing inflammatory process along with an alteration of hepatic lipid metabolism [28,29]. All the findings may suggest an important role for TXNDC5 in hepatic steatosis development. 

Recent evidence has found that in vivo deletion of *Txndc5* in endothelial cells has been shown to increase endothelial nitric oxide synthase protein and reduce atherosclerosis in Apoe-deficient mice [30]. Moreover, TXNDC5 promoted liver fibrosis through redox-dependent hepatic stellate cell activation [31], but its inhibition alleviated LPS-induced sepsis by inhibiting the NF-kappaB signaling pathway [32]. These recent advances suggest that TXNDC5 may have an impact role on hepatic transcriptome as well. In this manuscript, we developed *Txndc5*-deficient mice using CRISPR-Cas9 technology and performed a liver RNA-seq to characterize and elucidate if any of the hepatic steatosis factors may be linked with this PDI. 

## 2. Materials and Methods

### 2.1. Generation of Txndc5-Deficient Mice Using CRISPR/hifiCas9 Genome-Editing

Recombinant high fidelity (hifi) Cas9 enzyme and two single guide RNAs (sgRNA) targeting intron 2 and intron 3 of *Txndc5* were electroporated into C57Bl/6J mouse zygotes to generate mice with a deletion spanning exon 3 of *Txndc5* (Supplemental Figure S1A). Briefly, the two CRISPR RNA guides were designed using CHOPCHOP (http://chopchop.cbu.uib.no/; accessed on 5 May 2018) and were used as annealed two-part synthetic crRNA and tracrRNA molecules (Alt-R^®^ CRISPR guide RNAs, Integrated DNA Technologies, Inc. (IDT), Leuven, Belgium).

The resulting sequences were crRNA1: 5′-CCAACCAGAAAGGGCACAAG-3′ and crRNA2: 5′-CCAACCAGAAAGGGCACAAG-3′. The ribonucleoprotein mixes were prepared as described [33]. The hifi Cas9 protein (IDT #1081060) in complex with both targeting sgRNA (cr:trac RNA) (IDT #1072532) that direct the Cas9 at both ends of the third exon of *Txndc5* were electroporated into C57BL/6J (Janvier labs, Le Genest-Saint-Isle, France) mouse zygotes in M2 medium and cultured overnight at 37 °C [33]. Two-cell embryos were transferred into the uterus of pseudopregnant Swiss (Janvier labs) female mice at 2.5 dpc. The preparation of mouse zygotes, electroporation of hifi Cas9/sgRNAs, embryo transfer, and initial breeding of the *Txndc5*-deficient mice were performed by the transgenic mouse facility at the Centro de Investigación Biomédica de Aragón (CIBA) as previously described [34]. Founders harboring a deletion of the intended target site were identified by the presence of a smaller PCR amplicon (~132 base pair) instead of the original amplicon (432 bp), corresponding to the expected size of the region with exon 3 deleted using 5-forward primer 5′-GCAGCTATGCTATGTTCTTGAGCT-3′ and reverse primer 5′-GCATGGTTTTTGTTACCTCATTGG-3′. Four different founders with the deletion were obtained of 31 born mice from electroporated zygotes, all PCR products of the founders were confirmed by sequencing.

The potential off-targets of CRISPR were assayed by sequencing analysis at off-target sites predicted by CHOPCHOP, CRISPRater and Off-spotter. Four loci displaying the highest likelihood of unspecific cleavage of each guide were selected and amplified on the first mice generation and T7 endonuclease assay (#IC006, Genecopoeia, Rockville, MD, USA) was used to check off-target effects. 

One random male founder was crossed to C57BL/6J wild type mice to obtain heterozygous offspring; the heterozygous progeny was crossed to generate homozygous *Txndc5*-deficient mice (C57BL/6JRj-*Txndc5*<em2Mdnp>/Ciba, MGI:6444222). The latter were born at the expected Mendelian frequency, showed no detectable developmental defects, thrived at normal rate and both sexes were fertile.

### 2.2. Animals and Diets

Two-month-old male mice were used in all experiments and housed in sterile filter-top cages on a 12-h light/12-h dark cycle at the CIBA. All had *ad libitum* access to food and water. Mouse experiments were carried out in accordance with the EU Directive 2010/63 on the protection of animals used for scientific purposes and the study protocol was approved by the Ethics Committee for Animal Research of the University of Zaragoza (PI35/18 and PI03/21).

For 4 weeks, mice received a purified diet, based on the purified AIN-93 diet for laboratory mice. All diets were prepared in our facilities, lyophilized, and stored at −20 °C until use. The composition of these diets was described previously [35]. Intake and body weights were monitored every week. At the end of the 4-week dietary intervention, food was withdrawn for 16 h, and the mice were weighed and then sacrificed by suffocation in a CO_2_ chamber. Blood samples were drawn by cardiac puncture, and plasma and serum were centrifuged at 3000× *g* for 10 min. The livers were removed and frozen in liquid nitrogen and stored at −80 °C until processing and an aliquot was stored in buffered formaldehyde.

### 2.3. RNA Extraction

Each liver was homogenized using Tri Reagent from Ambion^®^ (Life Technologies, Carlsbad, CA, USA) and total RNA was extracted using spin column kit Direct-zol^TM^ RNA Miniprep (Zymo Research, Irvine, CA, USA), following the manufacturer’s instructions. RNA was quantified by absorbance at A_260/280_ using Nanodrop Spectrophotometer (Thermo) and the ratio was greater than 1.75 as well as the ratio A_260/230_. The integrity of the 28S and 18S ribosomal RNAs was verified by 1% agarose gel electrophoresis followed by ethidium bromide staining with a ratio 28S/18S greater than 2.

### 2.4. RNAseq Analysis

For RNA sequencing, 3 pools of wild type mice were prepared using equal amounts of hepatic total RNA of three mice. Another 3 pools were prepared for *Txndc5*-deficient mice combining total RNA from three mice per pool. The resulting 6 samples were sent to the Beijing Genomics Institute (BGI Genomics, Shenzhen, China) service. RNA quality tested, library construction, sequencing reads and posterior clean, genome mapping, analysis, identification, and quantification were realized as previously described [36]. A Bioinformatics flow of about 4.52 Gb per sample had an average genome mapping rate of 93.1%. The complete datasets were deposited in the GEO database (Accession number GSE185515)**.**

### 2.5. Quantification of mRNA and cDNA Synthesis

To verify the most relevant changes induced by the absence of TXNDC5 using RNAseq, represented by signal log_2_ ratio > 1.0 or <−1.0 and a *p* value < 0.005 for upregulated and downregulated, respectively, 10 genes fulfilling these criteria were chosen. Primer design (Supplementary Table S1), RT-PCR and cDNA synthesis were carried out as previously described [36]. ViiA7 Real-TIME PCR System (Life Technologies) was used, and relative amount of mRNA was calculated using the comparative 2^−ΔΔCq^ method and normalized to the reference *Ppib* expressions.

### 2.6. Western Blotting

Liver protein extraction, quantification and transference to a PVDF membrane was done as previously described [37]. Rabbit polyclonal antibodies were used against the different proteins: TXNDC5 (#19834-1-AP) (1/1000) from Proteintech (Manchester, UK) and serum amyloid protein (# PA5-102456) (1/500) from Thermo Fisher Scientific (Madrid, Spain). Equal loadings were confirmed by using a mouse monoclonal anti- β-ACTIN (#A5441) (1/2000) from Sigma (St Louis, MO, USA). Membranes were washed three times with a PBS buffer containing 0.1% Tween 20 and incubated for 1 h at room temperature with conjugated goat anti-rabbit IgG (H&L) DyLight 800 secondary antibody (SA5-35575) and goat anti-mice IgG (H&L) DyLight 680 secondary antibody (SA5-35518), both from Thermo-scientific, diluted 1/80,000. Images were captured using an Odyssey^®^Clx (LI-COR, Bad Homburg, Germany).

### 2.7. Histological Analysis

A portion of the liver was fixed in formaldehyde, paraffin-embedded, and sectioned. Sections were then stained with hematoxylin and eosin, scanned and scored for lipid droplet area, inflammation, necrosis, and fibrosis by trained histologists blinded to the experimental groups. The lipid droplet areas were estimated in sections with Adobe Photoshop CS3 (Adobe Inc., San Jose, CA, USA) and expressed as percentage of total liver section as previously described [26].

### 2.8. Plasma Determinations and Liver Lipids

Total plasma cholesterol and triglyceride concentrations were measured in a microtiter assay, using Infinity^TM^ commercial kits (Thermo Scientific, Madrid, Spain). Plasma ketones were measured using a colorimetric assay (Fujifilm Wako chemicals, Richmond, VA, USA). Total apolipoprotein A1 (APOA1) and apolipoprotein A4 (APOA4) were quantified by ELISA using anti-mouse APOA1 (#K23001R, Biodesign, Standford, CA, USA) and anti-goat APOA4 (sc-19036, Santa Cruz Biotechnology, Heidelberg, Germany). Serum amyloid A protein was determined by an ELISA kit (#MOFI00094, ARP American Research Products, Inc.^TM^, Waltham, MA, USA). Serum arylesterase activity of paraoxonase (PON1) was assayed, as previously described [38], and PAH-AH activity was determined using PAF acetylhydrolase assay kit (#760901, Cayman Chemical, Ann Arbor, MI, USA). Malondialdehyde (MDA) was determined using Lipid Peroxidation (MDA) Assay Kit (#MAK085, Sigma).

Plasma lipoprotein profile was determined in 100 μL of pooled plasma samples from each group by fast protein liquid chromatography (FPLC) gel filtration using a Superose 6B column (GE Healthcare, Chicago, IL, USA) in 48 fractions as previously described [39].

Total cholesterol was determined using 0.01 mg/mL Amplex Red (#C291, Tebu-bio, Barcelona, Spain), 2 U/mL horseradish peroxidase, 0.5 U/mL cholesterol oxidase and 0.5 U/mL cholesterol esterase (#P8375, #228250, #C1403, Sigma) dissolved in 50 mM K_2_HPO_4_ and 25 mM CaCl_2_ pH 7.4. To determine non-esterified cholesterol, the same protocol without the addition of cholesterol estearase was used, and the difference between both was calculated and expressed as the esterified cholesterol. Phosphatidylcholine was determined using 0.01 mg/mL Amplex Red (#C291, Tebu-bio), 2 U/mL horseradish peroxidase, 0.5 U/mL choline oxidase and 0.5 U/mL phospholipase D (#P8375, #C5896, #P0065, Sigma) dissolved in 25 mM Tris/HCL and 25 mM CaCl_2_, pH 8, resorufin was measured at 550 nm excitation wavelength and 590 nm emission wavelength.

Hepatic lipids were extracted using chloroform-methanol, dried, solubilized in isopropanol and measured using Infinity^TM^ commercial kits (Thermo Scientific, Madrid, Spain).

### 2.9. Reactive Oxygen Species (ROS) Content in Lipoproteins

The presence of ROS was assessed by measuring the conversion of 2,7-dichlorofluorescein diacetate into fluorescent dichlorofluorescein [40] in FPLC-isolated fractions corresponding to the different lipoproteins [41]. Briefly, high density lipoproteins (HDL) were incubated at 37 °C with 10 µg of dichlorofluorescein, 0.1% sodium azide in PBS. After 2 h of incubation, fluorescence was measured at an excitation wavelength of 485 nm and an emission wavelength of 535 nm [41].

### 2.10. Statistics

Results are presented as means and their standard deviations. The normal distribution of data was analyzed according to Shapiro–Wilk test, and homology of variance among groups using Bartlett’s or Levene’s tests. Parameters fitting both criteria were analyzed using one-tailed Student’s *t* test. A Mann–Whitney U test or non-parametric Kruskal–Wallis ANOVA followed by Dunn’s multiple comparisons was used to compare the groups failing in any of the hypotheses. Association between variables was assessed by Spearman’s correlation coefficient (ρ). All calculations were performed using SPSS version 15.0 software (SPSS Inc, Chicago, IL, USA) or GraphPad Prism 5 for Windows (GraphPad, San Diego, CA, USA). A *p* value of less than 0.05 was considered statistically significant.

## 3. Results

### 3.1. Generation of Txndc5-Deficient Mice

We verified that the two-single guide RNAs designed to flank the third exon of *Txndc5* (Supplementary Figure S1A) produce the deletion of the exon, showing a 310 bp deletion which verified the correct deletion of the third exon of *Txndc5* (Supplementary Figure S1B). Off-target analyses were carried out in four potential loci for each guide, resulting in a non-specific cut (Supplementary Figure S1C). Lastly, the levels of expression at RNA and protein levels were verified, which allow us to confirm the total loss of TXNDC5 in the liver (Supplementary Figure S1D).

### 3.2. Somatometric Parameters

To avoid hormonal influences and due to a limited number of females, these were not included in this study, which represents a limitation 

**Figure 1:**
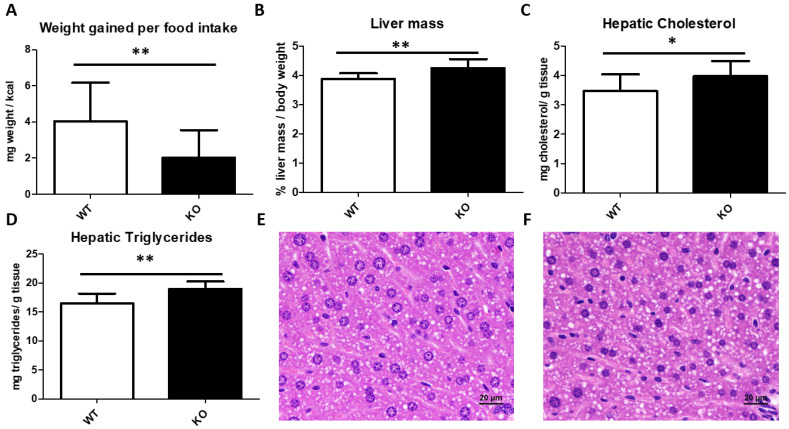
Somatometric parameters and hepatic fat content. Animal weight gained (mg) in 4 weeks per food intake (kcal) (**A**). Liver mass percentage of body weight after 16 h fasting (**B**). Hepatic cholesterol (**C**) and triglyceride (**D**) contents. Representative liver micrographs from wild type (**E**) and *Txndc5*-deficient mice (**F**), bar denotes 20 μm. Data are means ± SD for each group (n = 13 and n = 10, respectively, for WT and KO). Statistical analyses were done according to Mann–Whitney’s U-test and *, *p* value < 0.05; **, *p* value < 0.01.

For 4 weeks, 13 wild type (WT) and 10 *Txndc5*-deficient (or Knock-out, KO) two-month-old male mice born of heterozygous crosses were fed with a purified chow diet. After this period, KO males gained significantly less weight (mg) per kcal of food intake (Figure 1A). Mice were fasted 16 h prior to sacrifice, in which the percentages of loss of weight during fasting were not significantly different between both groups (5.8 ± 0.9 vs. 5.5 ± 1.2, *p* < 0.278 for WT and KO, respectively). In contrast with the animal weight, there was a significant increase in its liver weight (Figure 1B).

### 3.3. Liver Histological Analyses and Hepatic Lipid Content

According with the gained mass in KO livers, there was a significant increase in the hepatic content of both cholesterol and triglycerides (Figure 1C,D). However, the lipid droplet area did not differ between both groups, nor were there inflammatory foci (Figure 1E,F).

### 3.4. Hepatic Gene Expression of Txndc5-Deficient Male Mice Fed for 4 Weeks on a Chow Diet

To determine the impact of *Txndc5* on hepatic transcriptome, three RNA pools from 13 WT and 10 KO animals receiving the chow diet were submitted to next generation sequencing. Reads from each library were mapped onto the genome of reference followed by gene prediction, having a mapping ratio close to 93% (Supplementary Table S2). Transcripts were reconstructed, identifying 5419 novel transcripts, of which 4879 them were predicted coding transcripts. After genome mapping, the SNP and INDEL variant for each sample were analysed, with no difference observed between groups (Supplementary Table S3). Changes in the relative abundance of isoforms, regardless of the expression change, indicate a splicing-related mechanism. We detected five types of alternative splicing (AS) events, including Skipped Exon (SE), Alternative 5’ Splicing Site (A5SS), Alternative 3’ Splicing Site (A3SS), Mutually exclusive exons (MXE) and Retained Intron (RI), with no changes in total events (Supplementary Table S4).

**Figure 2:**
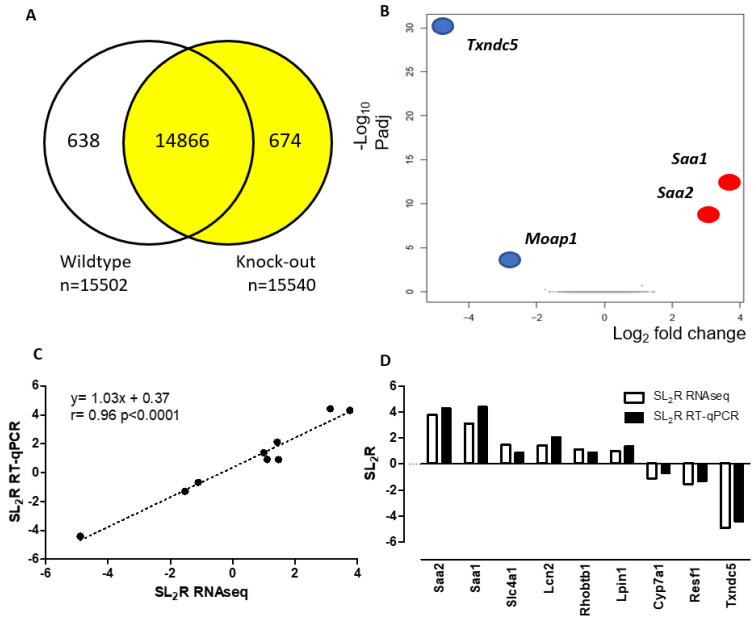
Differentially expressed genes. (**A**) Venn diagram of gene expression between groups. (**B**) Volcano plot of DEGs. X axis represents log_2_ transformed fold change. Y axis represents -log_10_ transformed significance. Red points represent upregulated DEGs. Blue points represent downregulated DEGs. Grey points represent non-regulated DEGs. Gray points represent genes with no changes. (**C**) Correlation analysis of 10 selected genes between RNAseq and RT-qPCR normalized to the invariant *Pipb* gene. The mean values obtained for signal log_2_ ratio (SL_2_R) from individual analyses were plotted against the RNAseq (Table 1). Good agreement between the procedures was seen (*r* = 0.96, *p* < 0.0001). (**D**) Changes in values of SL_2_R expression of both methods for the 10 selected genes.

Differentially expressed genes, shown in Figure 2A, were 638 in the WT and 674 in the KO group. Differentially expressed genes (DEGs) with <−2 or >2 expressed as log_2_ ratio and a low false discovery rate of *p* < 0.0001 displayed only 4 genes (Figure 2B).

To validate the RNAseq method, 10 genes with a signal log_2_ ratio > 1 or <−1 and a *p* value < 0.005 for upregulated and downregulated were randomly chosen to design their RT-qPCR assays (Table 1). The latter were carried out on individual hepatic RNA samples of each mouse. Using the log_2_ ratio of fold changes obtained by RNAseq and RT-qPCR for the ten selected transcripts, a correlation analysis was carried out with a significant agreement (*r* = 0.96, *p* < 0.0001) between both methods (Figure 2C) and all samples were properly categorized (Figure 2D).

All the genes selected, but *Cyp7a1*, were significantly regulated, with a heavily difference in the predicted genes in RNAseq (*Saa1*, *Saa2* and *Txndc5*). Two genes were found highly significantly regulated: *Lcn2*, a potential biomarker for hepatic steatosis, damage, and inflammation [42], and *Resf1*, a factor regulator of epigenetics modification associated with SETDB1 [43]. However, *Moap1* cannot be amplified by RT-qPCR due to its low expression, the number of its transcripts found in RNAseq being much fewer than the amplified genes.

### 3.5. Serum Amyloid Content on Liver and Plasma

**Figure 3:**
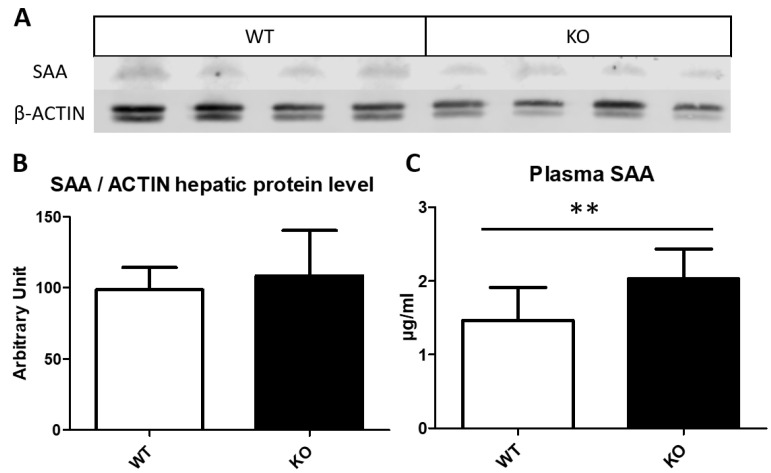
Serum amyloid A protein level. (**A**) Western blot of three protein pools from hepatic tissue in wild type (WT) and *Txndc5*-deficient (KO) male mice where SAA (13 kDa) and β-ACTIN, used as loading control, were detected. (**B**) Western blot bands were quantified using ACTIN as reference. (**C**) Total plasma SAA quantified by ELISA. Data are means ± SD for each group (n = 13 and n = 10, respectively, for WT and KO). Statistical analyses were done according to Mann–Whitney’s U-test and **, *p* value < 0.01.

Both serum amyloid A1 (SAA1) and A2 (SAA2) are acute phase complexed to HDL as apolipoproteins that are concurrently expressed in the liver, in response to inflammatory stimuli [44]. *Saa1* and *Saa2* genes are ~3.5 kb and codified 122 amino acid-long similar sequences which differed in only 9 positions [45]; moreover, these genes are thought to have been formed through gene duplication [44,46]. The increased expression of both genes at the mRNA level (Table 1) was reflected in a strong correlation (ρ = 0.948, *p* < 0.0001), between *Saa1* and *Saa2* expressions. These mRNA changes were suggestive of changes at the protein level. Surprisingly, when hepatic SAA protein levels were determined by Western blot (Figure 3A,B) no significant changes were observed; however, SAA showed a ~1.4-fold increase in the plasma of *Txndc5*-deficient mice (Figure 3C). Furthermore, a significant negative correlation was found in the WT group between *Txndc5* and plasma SAA (r = −0.566, *p* < 0.02), confirming the role of this PDI to control SAA expression. 

### 3.6. Plasma Parameters Determinations

Plasma basic parameters were measured and non-significant changes in the total plasma for triglycerides, cholesterol, phosphatidylcholine and ketone bodies were observed (Table 2).

**Figure 4:**
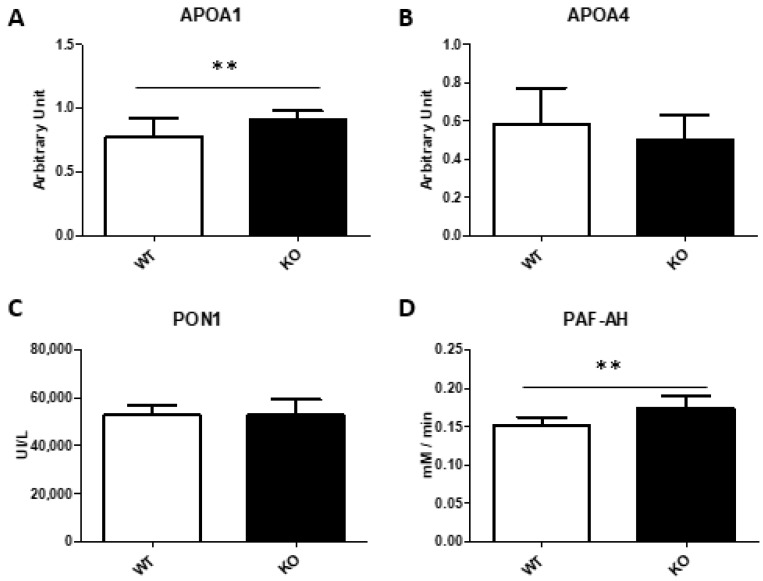
Influence of Txndc5 and SAA in functional plasma parameters. APOA1 (**A**) and APOA4 (**B**) were determined by ELISA while activity assays were done to determine paraoxonase 1 (**C**) and PAF-acetylhydrolase activities (**D**). All measurements were done in total serum collected from mice fed for 4 weeks on a chow diet and fasted 16 h prior to the sacrifice. Data are means ± SD for each group (n = 13 and n = 10, respectively, for WT and KO). Statistical analyses were done according to Mann–Whitney’s U-test and **, *p* value < 0.01.

As SAA tends to be accumulated in high density lipoproteins (HDL) as minor components, we determined the concentration of two proteins of HDL and apolipoprotein 1 and 4 (APOA1 and APOA4) to elucidate whether the presence of the amyloid may have an impact on the HDL protein composition. Their protein concentrations showed no changes in APOA4 but a surprising augment of APOA1 in *Txndc5*-deficient mice (Figure 4A,B).

One crucial aspect of TXNDC5 is its antioxidant properties involved in several processes; the determination of malondialdehyde (MDA) is commonly used as a marker for oxidative stress of lipid peroxidation that occurs because of oxidative damage [47]. Consequently, a significant increase in plasma MDA was seen in *Txndc5*-deficient mice (Table 2), adding more evidence for the antioxidant role of TXNDC5.

As there was an increased plasma lipid peroxidation, the antioxidant enzymes of HDL were assayed. In this sense, serum paraoxonase 1 (PON1) activity, an esterase with cardioprotective properties involved in several human diseases [48,49] and linked to APOA1, showed no change by the inactivation of the *Txndc5* gene (Figure 4C). By contrast, the activity of PAF-acetylhydrolase (PAF-AH), whose activity has been found to hydrolyse oxidized phospholipids [50], was increased in *Txndc5*-deficient mice (Figure 4D). In addition, PAF-AH activity and APOA1 were highly correlated (ρ = 0.748, *p* < 0.0001), showing an association between these parameters not influenced by the inactivation of the *Txndc5* gene.

### 3.7. Analysis of Lipoproteins

**Figure 5:**
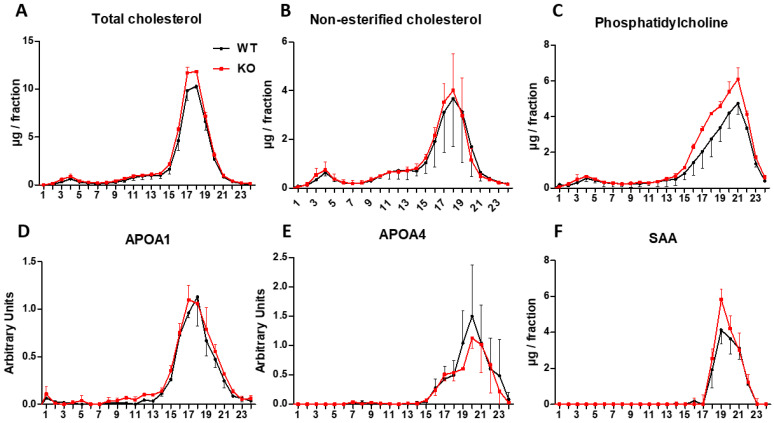
Influence of *Txndc5* deficiency on plasma FPLC chromatographs of male mice on a chow diet. Mice were 4 weeks on a chow diet and fasted 16 h prior sacrifice. Two pools per experimental group were prepared and data are represented as mean ± SD. Fluorometric assays were performed to determine total cholesterol (**A**), non-esterified cholesterol (**B**) and phosphatidylcholine (**C**). ELISA assays were used to measure APOA1 (**D**), APOA4 (**E**) and SAA (**F**).

Two pools of plasma for each group were prepared and separated by FPLC. Total cholesterol, non-esterified cholesterol and phosphatidylcholine were determined along with protein levels of APOA1, APOA4 and SAA to elucidate if the increase of SAA and APOA1 might influence the size and content of HDL. No difference was observed in the distribution of the measured parameters (Figure 5), cholesterol being mainly carried in HDL particles, which can be divided into two different populations of HDL: the large HDL loaded with more cholesterol (lHDL) and the small population of nascent HDL (sHDL) with more phosphatidylcholine. While the APOA1 was mainly located in lHDL, SAA was found in the sHDL.

**Figure 6:**
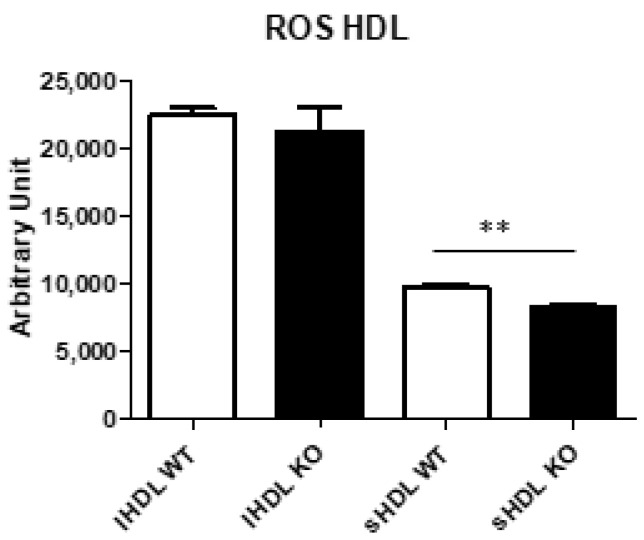
ROS content of HDL subgroups in wild type and *Txndc5*-deficient male mice. Two pools of each group were separated by FPLC and fractions 16–19 and 20–22 were combined into large HDL (lHDL) and small HDL (sHDL), respectively. Data are means ± SD for each group. Statistical analyses were done according to Mann–Whitney’s U-test and **, *p* value < 0.01.

To clarify whether SAA induced dysfunctional sHDL, two pools per group were prepared and PON1 and PAF-AH assayed. PON1 and PAF-AH activities were detected in both lHDL and sHDL, proving that SAA augment did not seem to affect their functionality (data do not show). To explore ROS content in lHDL and sHDL, DCF was assayed (Figure 6). There was a significant ROS reduction in sHDL of the *Txndc5*-deficient mice, but in this type of assay, it is hard to distinguish whether the lesser ROS signal is due to less oxidised lipids in the sample or to the antioxidant properties of APOA1 and PAF-AH preventing the ROS production.

## 4. Discussion

**Figure 7:**
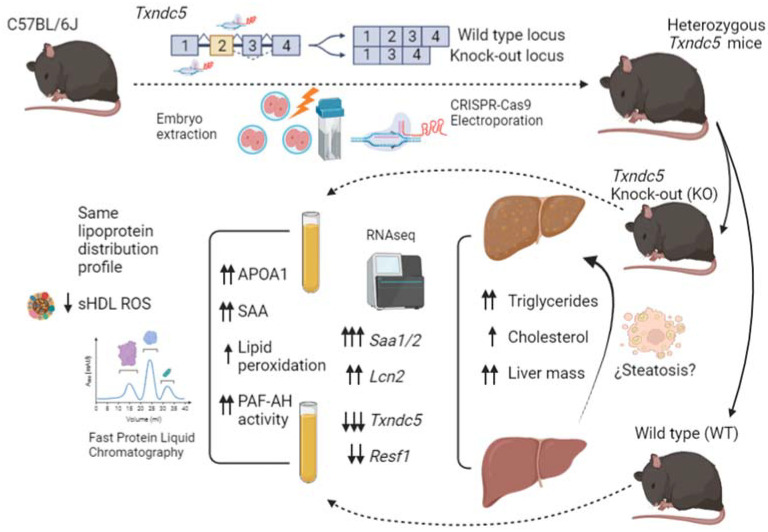
Comprehensive scheme displaying the experimental approaches. The preparation of knock-out mice, the analyses of livers and plasma and the main findings of hepatic RNAseq and plasma characterization are summarized.

This report describes the generation and phenotypic characterization of *Txndc5*-deficient mice in the terms represented in Figure 7. The latter showed an inefficient use of food to gain weight and an increased hepatic mass with higher contents of triglycerides and cholesterol. Next generation sequencing of the liver mRNA evidenced pronounced increases *Saa1* and *Saa2* expressions, verified by qPCR, along with *Lcn2* and *Resf1* changes. Amyloid augments were not reflected in the hepatic protein, but a discrete elevation of plasma SAA was observed, corresponding to small HDL. The characterization of small HDL showed a decreased ROS content in agreement with the increases of APOA1 and PAF-AH and in opposition to the increased plasma MDA levels (Table 2, Figure 4). Overall, TXNDC5 is a new player controlling the presence of SAA in HDL and this protein does not influence the antioxidant properties of these lipoparticles.

The inefficient use of food to gain weight as well as the increased hepatic mass have not been described in previous generations of *Txndc5*-deficient mice [51,52]. We may discard a potential malabsorption, considering that TXNDC5 deficiency resulted in hepatomegaly linked to increased triglyceride and cholesterol contents and normal values of these plasma parameters. Little is known regarding the regulation of hepatic *Txndc5* expression. In order to gain insight into this issue, we searched for submitted liver transcriptomes in the GEO database (Supplementary Table S5). Two studies reported changes associated with dietary fat content: GDS1307 and GDS1517. In the first one, four types of high fat diets were administered, differing in their fatty acid composition. The livers with the highest *Txndc5* expressions corresponded to the rats who received the fish oil diet, being the only group that did not show a hepatic triglyceride augment compare with the control group. In the second one, *Txndc5* was significantly increased in both wild type and *Scdl*-deficient mice on a low-fat and high-carbohydrate diet. In addition, in *Apoe*-deficient mice receiving squalene, we observed an inverse association between the hepatic lipid droplet area and *Txndc5* expression [8]. These facts suggest that TXNDC5 is involved in the fine-tuning of the lipid metabolism, but its effect is very subtle and more stressful conditions are required to observe an overt phenotype.

Using RNAseq, we have identified that *Txndc5* inactivation induced increased hepatic *Saa1* and *Saa2* expressions, resulting in a significant increase in plasma SAA (Figure 3). These results suggest a role of TXNDC5 in the regulation of these gene expressions in a setting of discrete hepatomegaly and absence of histological features of inflammation. In fact, plasma SAA levels were significantly correlated with the liver mass (ρ = 0.563, *p* < 0.005), but not with hepatic triglycerides. SAA is an acute phase protein produced in hepatocytes after induction by cytokines and released into the plasma linked to HDL in mice [46]. Surprisingly, the observed increase at the plasma level was rather modest compared with the magnitude of elevated mRNAs, suggesting an inefficient translation, a facilitated export into plasma or a rapid removal from this compartment. All these aspects have been described related to SAA [53]. The fact that hepatic protein did not experience any significant change may reinforce the first interpretation in agreement with the findings of Chait et al. [54]. Two genes were significantly regulated by *Txndc5* inactivation: Lipocalin 2 (*Lcn2*) and *Resf1* and their changes were found to correlate with the hepatic triglycerides as well as *Saa1* and *Saa2* expressions (*p* < 0.01). *Lcn2* is a potential biomarker for hepatic inflammation regulated by cytokines as well [42]. *Resf1* is a silencing factor, which regulates repressive epigenetic modifications associated with SETDB1 [43]. The analysis of genes regulated by several factors including SETDB1 identified pathways similar to those upregulated in old livers, that would contribute to metabolic dysfunction [55]. In this way, a reduction in *Resf1* could accelerate this genetic program of aging. PAF-AH activity has been also found to be modulated by cytokines and its activity helps to decrease not only lipid peroxidation but pro-inflammatory interleukins [56]. In the present report, the increased hepatic *Lcn2* expression together with the increased *Saa1*, *Saa2* expressions and PAF-AH activity seem to suggest an acute response in the absence of liver inflammation elicited by the lack of TXNDC5. A 16-h fasting is considered a stressor in mice fed *ad libitum*, losing around 5–6% of their body weight and activating their ketone metabolism and, consequently, fatty acid oxidation [57]. In our experimental approach, this fasting regime has been adopted, so it is reasonable to assume that the absence of TXNDC5 makes mice prone to develop acute phase responses. All these aspects have been described related to SAA [53]. Moreover, TXNDC5 is known to play an important role in inflammation and controlling redox status [10,58,59], its absence made cells are more susceptible to apoptosis in several tissues [21,23], and its expression has been correlated with bad prognosis in multiple types of cancer since it provides more ROS tolerance [10,13,60]. ROS control has been linked to TXNDC5 through JNK/STAT3 and ATF6 pathways, confirming the PDI function to modulate oxidative stress. ROS levels are related to aging and metabolic dysregulation [61] as *Resf1* down expression, along with biomarkers of hepatic inflammation (*Saa1/2* and *Lcn2*) that are significantly correlated with hepatic triglycerides, offers a vision of how the absence of Txndc5 has an impact on hepatic steatosis as a global multifactorial disease in which more studies need to be addressed. In this regard, there were increased hepatic triglyceride and plasma malondialdehyde levels, and these stressors might have induced the inflammatory cascade that triggered *Saa1* and *Saa2* expressions resembling a sterile inflammation. 

To elucidate whether SAA has an impact on plasma lipid metabolism, several plasma parameters were analyzed. A higher APOA1 concentration was observed, this main component of HDL highly correlated with the increased PAF-acetylhydrolase activity. In addition, the plasma SAA increase did not influence cholesterol distribution in lipoproteins since it was mainly carried out in HDL as it corresponds to C57BL/6J mice. SAA was found in the small HDL containing more phosphatidylcholine than the large HDL loaded with cholesterol and APOA1. The absence of changes in large HDL cholesterol suggests that SAA-containing small HDL particles are not interfering the reverse-cholesterol transport in agreement with previous reports [62]. The ROS content of both HDL subgroups showed no difference in large HDL and a lesser content in small HDL by TXNDC5 deficiency, the latter is consistent with the APOA1 and PAF-AH augments and their antioxidant properties. Increased levels of SAA are associated with an increased risk for atherosclerosis and are being considered as a predictor for acute coronary syndrome [63,64]. However, the absence of SAA did not reduce atherosclerosis in experimental models [65]. The findings of the present report may add new complexity to the discrepancy observed regarding SAA role in atherogenesis.

## 5. Conclusions

TXNDC5, as we hypothesized, was correlated with hepatic steatosis and redox control in the liver, showing an increased liver mass with a higher fat content linked to *Saa1* and *Saa2* expressions. This metabolic unbalance produced by the lack of TXNDC5 under fasting stress led to a prodromal stage of sterile inflammation, increasing lipid peroxidation and releasing SAA to the plasma, which was compensated by an enhanced activity of PAF-AH, linked with APOA1. Serum amyloid does not seem to impair HDL antioxidant function in this early stage. However, how the absence of TXNDC5 may influence SAA regulation under more stressful conditions like non-alcoholic fatty liver disease, fibrosis or atherosclerosis remains unclear. These represent fascinating endeavours to understand the link among serum amyloid and lipid oxidation and their role in hepatic steatosis and inflammation.

## Figures and Tables

**Table 1:** Hepatic transcripts differentially expressed by the lack of *Txndc5* at the level of signal log_2_ ratio < or >1 and *p* value < 0.005 in male mice according to RNAseq and RT-qPCR.

Name	GenBank	Biological Process	Gene Symbol	SL_2_R RNA seq	*p* Value RNA seq	WT	KO	SL_2_R qPCR
Serum Amyloid A2	NM_011314.3	Acute phase protein	*Saa2*	3.76	5.91 × 10^−17^	1.3 ± 1	26 ± 27 ***	4.31
Serum Amyloid A1	NM_009117.4	Acute phase protein	*Saa1*	3.13	6.41 × 10^−13^	1.1 ± 0.5	23 ± 24 ***	4.41
Solute Carrier Family 4 Member 1	NM_011403.2	Anion Exchange Protein	*Slc4a1*	1.47	1.80 × 10^−3^	1.6 ± 1.1	2.9 ± 1.8 *	0.90
Lipocalin 2	NM_008491.1	Iron-trafficking protein	*Lcn2*	1.43	1.08 × 10^−3^	1.5 ± 1.2	6.3 ± 7 **	2.11
Rho Related BTB Domain Containing 1	NM_001081347.1	Small GTPase of Rho family	*Rhobtb1*	1.11	7.12 × 10^−3^	1.1 ± 0.7	2.5 ± 2 *	0.91
Lipin 1	NM_001355598.1	Triglyceride synthesis	*Lpin1*	1.00	3.74 × 10^−3^	1.1 ± 0.6	2.9 ± 2 *	1.37
Cytochrome P450, family 7, subfamily a, polypeptide 1	NM_007824.3	Bile acid synthesis	*Cyp7a1*	−1.11	2.79 × 10^−3^	1.4 ± 0.8	0.9 ± 0.6	−0.68
Retroelement Silencing Factor 1	NM_001289662.1	Regulation of imprinted gene expression	*Resf1*	−1.53	1.24 × 10^−3^	1.2 ± 0.7	0.5 ± 0.6 **	−1.30
Thioredoxin Domain Containing 5	NM_145367.4	Disulfide isomerase	*Txndc5*	−4.89	2.77 × 10^−35^	1.1 ± 0.4	0.1 ± 0 ***	−4.41
Modulator of Apoptosis 1	NM_001142937.2	Receptor-dependent apoptosis	*Moap1*	−2.85	6.35 × 10^−8^	NA	NA	NA

Mice were 4 weeks on a chow diet and fasted 16 h prior to sacrifice. Data are means ± SD for each group (n = 13 and n = 10, respectively, for WT and KO) normalized to *Ppib* as reference gene. Statistical analyses were done according to Mann–Whitney’s U-test and *, *p* value < 0.05; **, *p* value < 0.01; ***, *p* value < 0.005 vs. WT.

**Table 2:** Plasma parameters of male wild type and *Txndc5*-deficient mice.

	Wild Type (n = 13)	Knock-Out (n = 10)
Triglycerides (mM)	1.8 ± 0.3	2 ± 0.5
Cholesterol (mM)	2.7 ± 0.4	3 ± 0.2
Phosphatidylcholine (mM)	4.2 ± 1.4	4.7 ± 1.8
Ketone bodies (mM)	1.7 ± 0.5	1.7 ± 0.4
Malondialdehyde (mM)	0.59 ± 0.17	0.67 ± 0.12 *

Mice were 4 weeks on a chow diet and fasted 16 h prior sacrifice. Data are means ± SD for each group. Statistical analyses were done according to Mann–Whitney’s U-test and *, *p* value < 0.05.

## Data Availability

Data is contained within the article and supplementary material.

## Supplementary Materials

The following are available online at https://www.mdpi.com/article/10.3390/biomedicines10030709/s1, Figure S1: Targeted deletion of Txndc5 locus. Table S1: Characteristics of primers used in RT-qPCR according to MIQE guidelines. Table S2: Sequence quality metrics and genome mapping of GSE185515. Table S3: Summary of SNP variant type of GSE185515. Table S4: Summary of splice variant type of GSE185515. Table S5: Changes in hepatic *Txndc5* expression.
