# Supplementary material for: Thioredoxin Domain Containing 5 Suppression Elicits Serum Amyloid A-Containing High-Density Lipoproteins
Source: Biomedicines. 2022 Mar 18;10(3):709. doi: 10.3390/biomedicines10030709 (PMC8945230; doi:10.3390/biomedicines10030709)
Supplement: Supplementary file 1 [file biomedicines-10-00709-s001.zip › S Tables Thioredoxin domain containing 5 suppression elicits serum amyloid A-containing high density lipoproteins .pdf]

Table S1: Characteristics of primers used in RT-qPCR according to MIQE guidelines.

Gene symbol	Accession	Sequence	Amplicon length	Exons	[Primer]	Efficiency
Cyp7a1	NM_007824.3	Sense CATTACAGAGTGCTGGCCAAGA<br>Antisense CTGTCCGGATATTCAAGGATGC	144	4/5	0.1 µM	97%
Lcn2	NM_008491.1	Sense GGAACGTTTCACCCGCTTTG<br>Antisense TGAACCATTGGGTCTCTGCG	140	5/6	0.1 µM	104%
Lpin1	NM_001355598.1	Sense TACCCCCAGTCAGCATCGTA<br>Antisense CTTTTTGGTGTTGAGGGCCG	89	5/6	0.1 µM	96%
Resf1	NM_001289662.1	Sense AATCCCGACGGAGGGACTAA<br>Antisense GCTTTGGAGGTGGCCTGTAT	232	3	0.1 µM	98%
Rhobtb1	NM_001081347.1	Sense CGGGAGCGAGAGCTTTGTAA<br>Antisense CAGCAGCTGGTACTGTGTCA	158	2/3	0.1 µM	101%
Saa1	NM_009117.4	Sense TTAGCTCAGTAGGTTGTGCTGG<br>Antisense ACAATGTTTCCCCAGAGAGCA	92	4	0.1 µM	97%
Saa2	NM_011314.3	Sense CTGGCTGGAAAGATGGAGAC<br>Antisense TGTCCTCGTGTCCTCTGC	147	3/4	0.1 µM	104%
Slc4a1	NM_011403.2	Sense GATCCCAGATCGAGACAGCG<br>Antisense CACATAGACCTGACCGGAGC	184	3/5	0.1 µM	107%
Txndc5	NM_145367.4	Sense CAGGCTTGTCAGATGTCACCAT<br>Antisense TAACCTCGTACCGAGTACTTGCTG	82	8/9	0.2 µM	100%
Ppib	NM_011149	Sense GGAGATGGCACAGGAGGAA<br>Antisense GTAGTGCTTCAGCTTGAAGTTCTCAT	72	3/4	0.1 µM	103%

Table S2: Sequence quality metrics and genome mapping of GSE185515.

Group	Total Raw Reads (M)	Total Clean Reads (M)	Clean Reads (%)	Mapping Ratio (%)
Wildtype (n=3)	48,31 ± 1,44	44,69 ± 1,08	44,69 ± 1,08	92,86 ± 1,0652
Knock-out (n=3)	49,97 ± 1,44	45,73 ± 1,08	45,73 ± 0,36	93,34 ± 0,42

Supplementary Table S3: Summary of SNP variant type of GSE185515.

Group	A-G	C-T	Transition	A-C	A-T	C-G	G-T	Transversion	Total
Wildtype	1673 ± 342	1677 ± 351	3350 ± 692	365 ± 93	336 ± 64	301 ± 80	319 ± 78	1321 ± 306	4670 ± 989
Knock-out	1621 ± 342	1635 ± 351	3256 ± 692	350 ± 93	277 ± 64	297 ± 80	328 ± 78	1252 ± 306	4509 ± 989

A-G: The amount of A-G variant type; C-T: The amount of C-T variant type; Transition: The amount of A-G and C-T variant type; A-C: The amount of A-C variant type; A-T: The amount of A-T variant type: C-G: The amount of C-G variant type; G-T: The amount of G-T variant type; Transversion: The amount of A-C, A-T, C-G and G-T variant type Total: The amount of all variant type

Supplementary Table S4: Summary of splice variant type of GSE185515.

Group	SE	MXE	A5SS	A3SS	RI
Wildtype	3353 ± 1019	275 ± 85	693 ± 122	831 ± 150	338 ± 54
Knock-out	3508 ± 1019	302 ± 85	718 ± 122	905 ± 150	346 ± 54

SE: Skipped Exon, MXE: Mutually exclusive exons, A5SS and A3SS: Alternative 5' or 3′Splicing Site respectively and RI: Retained Intron.

Supplementary Table S5. Changes in hepatic *Txndc5* expression.

Experimental condition	Type of change	Signal $\log_2$ ratio	Accession number
Fish oil	Increased	0.6	GDS1307
Iron deficient, LPS injected mice	Increased	0.5	GDS5271
TGFβ-activated kinase 1 deficiency	Increased	0.5	GDS5474
Very low-fat diet in mice	Increased	0.3	GDS1517
Alcoholic hepatitis	Increased	0.1	GDS4389
Dexamethasone	Decreased	-0.6	GDS5035
NF-E2-related factor 2 Nrf2 deficiency	Decreased	-0.3	GDS3476
Sphingosine 1-phosphate lyase deficiency	Decreased	-0.12	GDS3654
Caspase-1 deficiency	No change	-0.06	GDS4922
Dicer1 deficiency	No change	-0.05	GDS3685
Steroid receptor coactivator-2 deficiency	No change	0.05	GDS4785
Interleukin 6	No change	0.02	GSD3773

Data obtained from Genome Expressed Omnibus data bank and Array express. https://www.ncbi.nlm.nih.gov/geoprofiles/ https://www.ebi.ac.uk/arrayexpress/.
