# Supplementary material for: Thioredoxin Domain Containing 5 Suppression Elicits Serum Amyloid A-Containing High-Density Lipoproteins
Source: Biomedicines. 2022 Mar 18;10(3):709. doi: 10.3390/biomedicines10030709 (PMC8945230; doi:10.3390/biomedicines10030709)
Supplement: Supplementary file 1 [file biomedicines-10-00709-s001.zip › S1 Figure Thioredoxin domain containing 5 suppression elicits serum amyloid A-containing high density lipoproteins.pptx]

## Slide 1
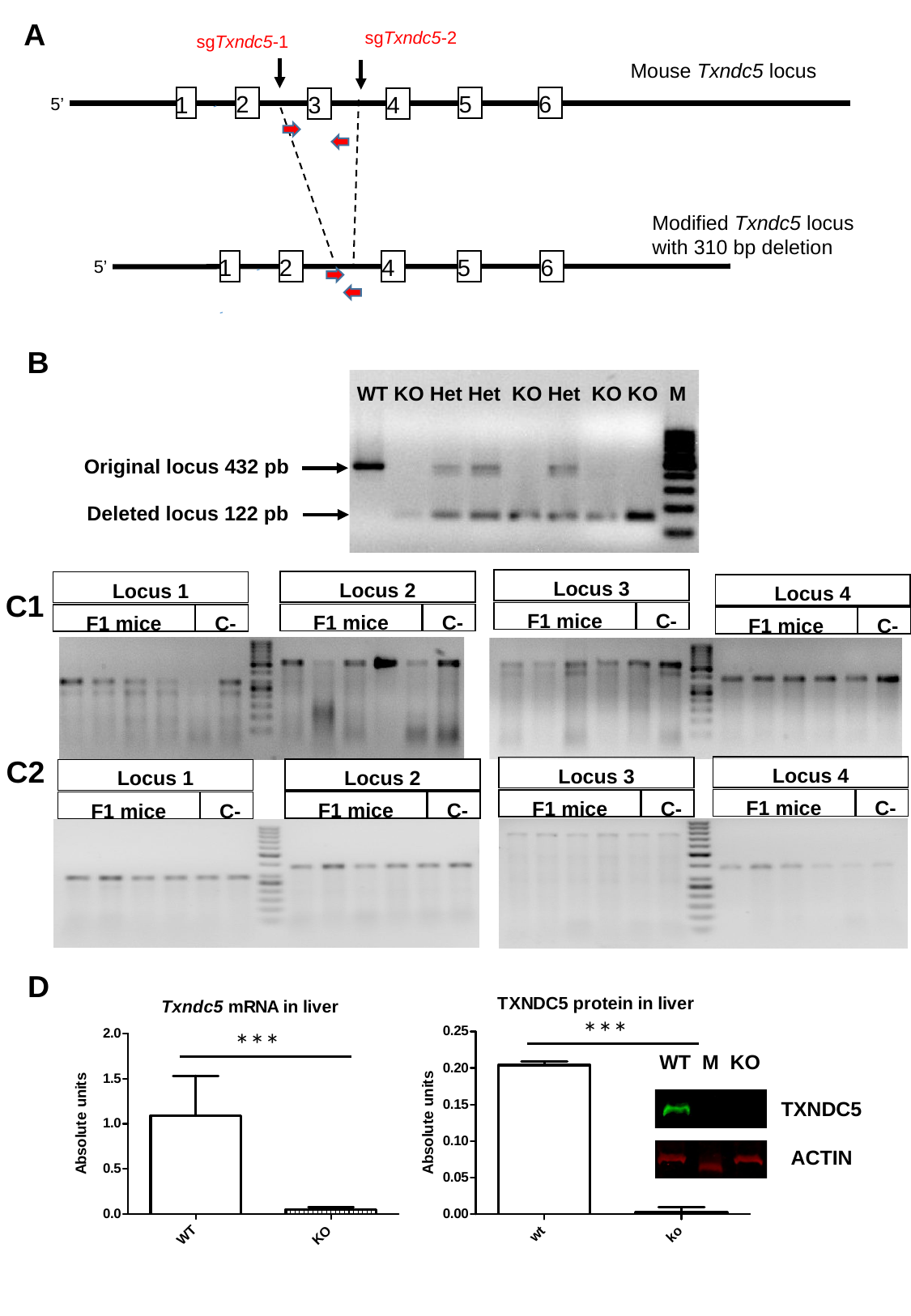

A
sgTxndc5-2
sgTxndc5-1
Mouse Txndc5 locus
6
2
5
4
3
1
5’
Modified Txndc5 locus with 310 bp deletion
6
1
2
4
5
5’
B
WT KO Het Het KO Het KO KO M
Original locus 432 pb
 Deleted locus 122 pb
Locus 3
F1 mice
 C-
Locus 2
F1 mice
 C-
Locus 1
F1 mice
 C-
Locus 4
F1 mice
 C-
C1
C2
Locus 4
F1 mice
 C-
Locus 3
F1 mice
 C-
Locus 2
F1 mice
 C-
Locus 1
F1 mice
 C-
D
***
***
WT M KO
TXNDC5
ACTIN
